# Familial severe non-ischemic dilated cardiomyopathy with coexisting likely pathogenic BAG3 and FLNC variants: a case report

**DOI:** 10.3389/fcvm.2026.2000926

**Published:** 2026-10-30

**Authors:** Wang Lili, Wang Youcao, Yao Xiaodong, Wang Zhen

**Affiliations:** Department of Cardiology, The Affiliated Hospital of Shandong University of Traditional Chinese Medicine, Jinan, Shandong, China

**Keywords:** BAG3, dilated cardiomyopathy, family screening, FLNC, heart failure, inherited cardiomyopathy, myocardial fibrosis, sanger sequencing

## Abstract

**Background:**

Dilated cardiomyopathy (DCM) is genetically heterogeneous. In severe non-ischemic DCM with familial clustering of heart-failure deaths, genetic testing and pedigree analysis may clarify etiology and guide cascade screening.

**Case presentation:**

A 40-year-old woman presented with progressive dyspnea, chest tightness, and palpitations after an upper respiratory tract infection. Echocardiography showed severe left ventricular systolic dysfunction [left ventricular ejection fraction (LVEF) approximately 20%], left atrial enlargement, functional mitral regurgitation, and a small pericardial effusion. Serial cardiac troponin I and myocardial enzyme measurements remained within the reference ranges. Coronary angiography excluded obstructive coronary disease. Cardiovascular magnetic resonance (CMR) demonstrated global left ventricular hypokinesis and a clinically reported non-ischemic late-gadolinium-enhancement pattern at the papillary-muscle level involving the anterior/lateral wall. Because cardiac biomarkers remained normal and dedicated inflammatory CMR sequences were not acquired, active myocarditis could not be confirmed. Guideline-directed therapy reduced NT-proBNP from 4154 to 828 pg/mL, although angiotensin receptor-neprilysin inhibitor therapy was limited by symptomatic hypotension. At 2 months, symptoms and daily activity tolerance improved, but LVEF remained 25%, QTc was 479 ms, and ventricular premature complexes persisted. Whole-exome sequencing identified heterozygous likely pathogenic variants in BAG3 (NM_004281.4:c.180 + 1G > T) and FLNC [NM_001458.5:c.761_779del, p.(Leu254Profs*14)]. The daughter carried the BAG3 variant, whereas both children were negative for FLNC. The proband reported heart-failure-related deaths in her father and younger brother during adulthood and in a nephew during childhood.

**Conclusions:**

The findings support complex familial non-ischemic DCM with marked variable expressivity. Coexisting BAG3 and FLNC variants should be interpreted as hypothesis-generating rather than proof of dual-gene causality. The infection-associated acute decompensation, persistently normal cardiac injury biomarkers, non-ischemic LGE, and incomplete inflammatory CMR characterization favor cautious interpretation rather than a definitive diagnosis of myocarditis. Serial imaging, rhythm surveillance, individualized device-risk assessment, and follow-up of genotype-positive relatives are essential.

## Background

Dilated cardiomyopathy (DCM) is defined by left ventricular or biventricular dilation and systolic dysfunction not explained by abnormal loading conditions or coronary artery disease. Contemporary evaluation integrates family history, electrocardiography, echocardiography, cardiovascular magnetic resonance (CMR), coronary assessment, and genetic testing, particularly in early-onset or familial disease (1–5).

Loss-of-function BAG3 variants cause autosomal dominant DCM through haploinsufficiency and impaired cardiomyocyte proteostasis, whereas truncating FLNC variants are associated with dilated/arrhythmogenic cardiomyopathy, myocardial fibrosis, and ventricular arrhythmias (3, 4, 6–8). Coexisting variants in robust cardiomyopathy genes require cautious interpretation unless segregation or functional evidence demonstrates additive pathogenicity (5, 9, 10).

We describe severe familial non-ischemic DCM with an acute heart failure presentation following an upper respiratory tract infection and coexisting likely pathogenic BAG3 and FLNC variants.

## Case presentation

### Initial presentation and diagnostic evaluation

A 40-year-old woman was admitted on March 31, 2026, with 20 days of episodic chest tightness and dyspnea that worsened over 5 days after an upper respiratory tract infection. Symptoms included palpitations, cough, orthopnea, abdominal distension, fatigue, and exercise intolerance. She had no hypertension, diabetes, chronic kidney disease, or known prior cardiac disease.

Temperature was 36.3 °C, heart rate 103 beats/min, respiratory rate 20 breaths/min, and blood pressure 118/91 mmHg. Examination showed coarse breath sounds and mild bilateral leg edema. Initial echocardiography demonstrated LVEF approximately 20%, left atrial enlargement, mild-to-moderate mitral regurgitation, mild tricuspid and aortic regurgitation, and a small pericardial effusion. Chest computed tomography showed pulmonary edema, bilateral pleural effusions, cardiomegaly, and pericardial effusion. NT-proBNP was 6990.23 pg/mL at the referring hospital.

In hospital, LVEF was approximately 21%. NT-proBNP decreased from 4,154 pg/mL on April 1 to 1,094 pg/mL on April 6 and 828 pg/mL on April 12. Serial cardiac troponin I and myocardial enzyme measurements were obtained on multiple occasions during hospitalization and remained within the respective reference ranges, without biochemical evidence of acute myocardial necrosis. Antinuclear antibody was positive at 1:100 (speckled), but rheumatology found insufficient evidence for systemic connective tissue disease. Transient liver-enzyme elevation improved with decongestion.

### Imaging, rhythm assessment, treatment, and follow-up

Coronary angiography on April 2 showed a left-dominant circulation without significant stenosis. CMR on April 9 demonstrated global left ventricular hypokinesis, left atrial enlargement, mild mitral regurgitation, a small pericardial effusion, and a clinically reported non-ischemic late-gadolinium-enhancement pattern at the papillary-muscle level involving the anterior/lateral wall. The examination did not include dedicated inflammatory sequences such as T2-weighted imaging, T2 mapping, or STIR. Given the repeatedly normal cardiac troponin I and myocardial enzyme measurements and the absence of these inflammatory CMR sequences, active myocarditis could not be established; the episode was therefore interpreted as infection-associated acute decompensation in the setting of inherited cardiomyopathy. Echocardiography showed transient LVEF improvement to approximately 27% on April 10.

Serial electrocardiograms on March 31, April 2, and May 25 showed sinus rhythm, poor R-wave progression/QS complexes in the anterior precordial leads, repolarization abnormalities, and ventricular premature complexes on admission and follow-up. QTc values were 494, 463, and 479 ms, respectively. Standard 24-hour ambulatory Holter monitoring was not arranged during index hospitalization restricted by local medical insurance policies: simultaneous application of bedside continuous cardiac telemetry and 24-hour Holter would result in complete non-reimbursement of all cardiac monitoring charges. Therefore, only ward bedside telemetry was adopted for inpatient real-time rhythm surveillance, and quantitative analysis of total 24-hour ventricular ectopy burden could not be conducted (detailed ECG parameters summarized in Supplementary Table S1) (Figure 1).

**Figure 1:**
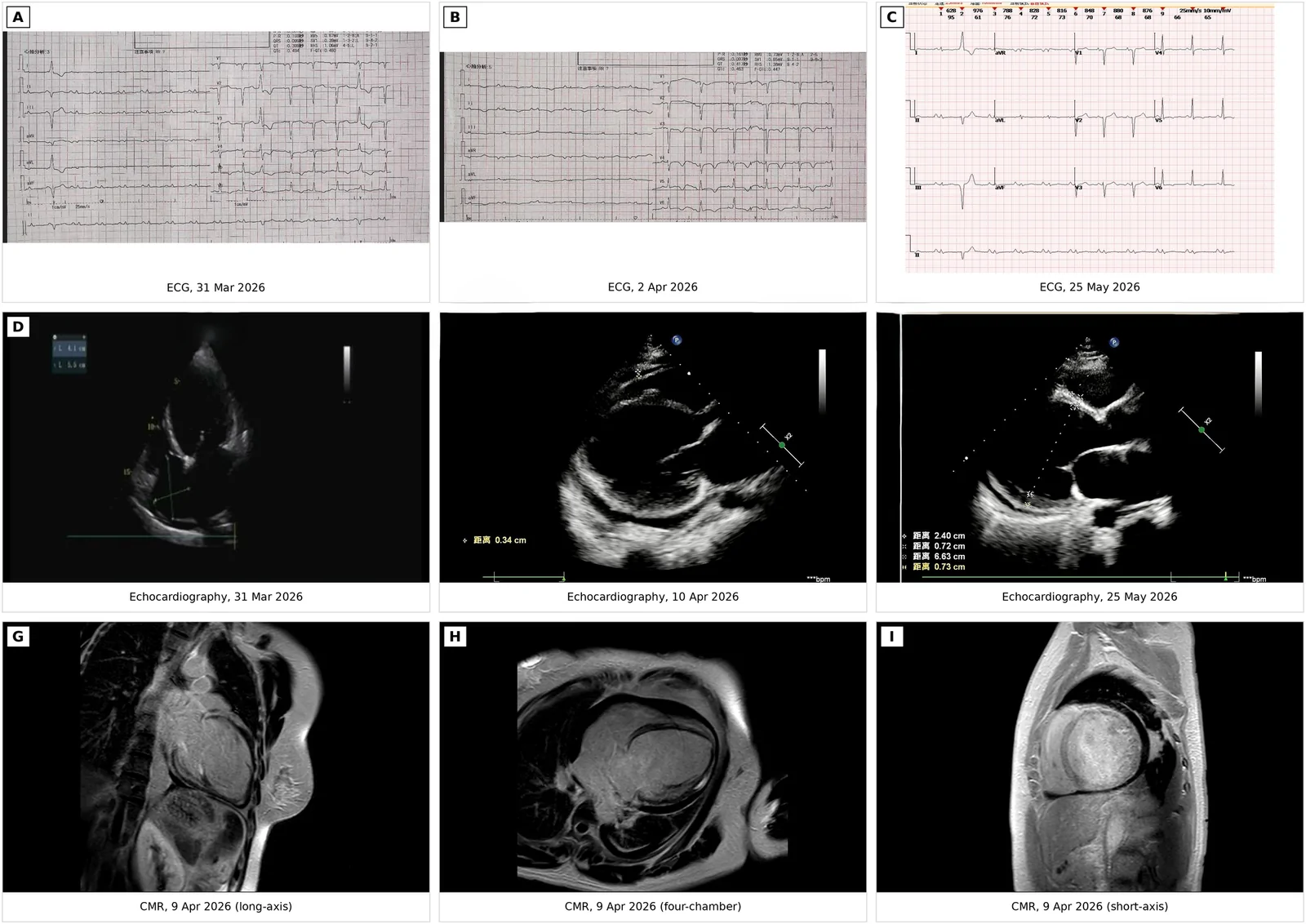
Serial de-identified electrocardiographic, echocardiographic, and cardiovascular magnetic resonance (CMR) findings. (A-C) Electrocardiograms recorded on March 31, April 2, and May 25, 2026, respectively; ventricular premature complexes are visible in panels A and C. (D-F) Echocardiograms obtained at presentation, on April 10, and on May 25, 2026, respectively, showing persistent left ventricular dilation and severe systolic dysfunction. (G-I) CMR images acquired on April 9, 2026; the clinical report documented global hypokinesis and a non-ischemic late gadolinium enhancement pattern at the papillary-muscle level involving the anterior/lateral wall. All images were de-identified before publication. Written informed consent for publication of the clinical images was obtained from the patient.

A-C, Electrocardiograms recorded on March 31, April 2, and May 25, 2026; ventricular premature complexes are present in A and C. D-F, Echocardiograms obtained at presentation, on April 10, and on May 25, showing persistent left ventricular dilation and severe systolic dysfunction. G-I, CMR images acquired on April 9; the clinical report documented global hypokinesis and non-ischemic late gadolinium enhancement at the papillary-muscle level involving the anterior/lateral wall. All clinical images were fully de-identified before publication, and all direct patient identifiers were removed. Written informed consent for publication of these clinical images was obtained from the patient.

Treatment included loop diuretic, metoprolol succinate, spironolactone, dapagliflozin, and attempted sacubitril/valsartan. The angiotensin receptor-neprilysin inhibitor was stopped during hospitalization because of symptomatic hypotension and later reintroduced at low dose under outpatient blood-pressure monitoring. Other therapies were tolerated.

At 2-month follow-up, chest tightness had decreased and ordinary daily activities were tolerated. Heart rate was approximately 68 beats/min and systolic blood pressure approximately 100 mmHg. Echocardiography showed LVEF 25%, left atrial diameter 39 mm, right ventricular end-diastolic diameter 24 mm, estimated pulmonary artery systolic pressure 24 mmHg, mild mitral regurgitation, and a small pericardial effusion. Renal function remained stable; serum potassium increased from 3.36 to 4.22 mmol/L. The clinical course and key investigations are summarized in Table 1.

**Table 1:** Clinical timeline.

Date	Key clinical event
2026-03-30	Outside echocardiography: LVEF approximately 20%, left atrial enlargement, trace pericardial effusion, mild-to-moderate MR, mild TR, mild AR. Chest CT: interstitial pulmonary edema, bilateral pleural effusions, cardiomegaly, pericardial effusion. NT-proBNP 6990.23 pg/mL.
2026-03-31	Admission for chest tightness, palpitations, and progressive dyspnea after upper respiratory tract infection. Bedside echocardiography: LVEF approximately 21%. ECG: sinus rhythm with ventricular premature complexes and QTc 494 ms. Baseline renal and electrolyte tests performed.
2026-04-01	NT-proBNP 4,154 pg/mL; serial cardiac troponin I and myocardial enzyme measurements remained within reference ranges; ANA 1:100 (speckled pattern); rheumatology found insufficient evidence for systemic connective tissue disease; GDMT initiated.
2026-04-02	ECG: sinus rhythm with persistent conduction/repolarization abnormalities and QTc 463 ms. Coronary angiography: left-dominant circulation and no significant coronary stenosis. Continuous bedside telemetry, rather than formal 24-hour Holter monitoring, was used for inpatient rhythm surveillance.
2026-04-09	CMR: global LV hypokinesis, left atrial enlargement, mild MR, small pericardial effusion, and clinically reported non-ischemic late gadolinium enhancement at the papillary-muscle level involving the anterior/lateral wall.
2026-04-10	Repeat echocardiography: LVEF approximately 27%, with persistent left ventricular dilatation and severe systolic dysfunction.
2026-04-12	NT-proBNP decreased to 828 pg/mL; clinical improvement; pre-discharge ECG and safety laboratory testing performed.
2026-05-25	Two-month outpatient follow-up: chest tightness decreased and daily activities were tolerated; ECG showed sinus rhythm with ventricular premature complexes and the clinical report documented QTc 479 ms; echocardiography showed persistent left ventricular dilatation, LVEF 25%, mild MR, and a small pericardial effusion; renal function and electrolytes remained stable.
2026-05-31	Final genetic testing report issued after Sanger validation in the proband's son and daughter.

### Family history and pedigree

The proband reported heart-failure-related deaths in her father and younger brother during adulthood and in a nephew during childhood. This three-generation clustering suggests familial cardiomyopathy with marked variability in age at onset and severity (Figure 2).

**Figure 2:**
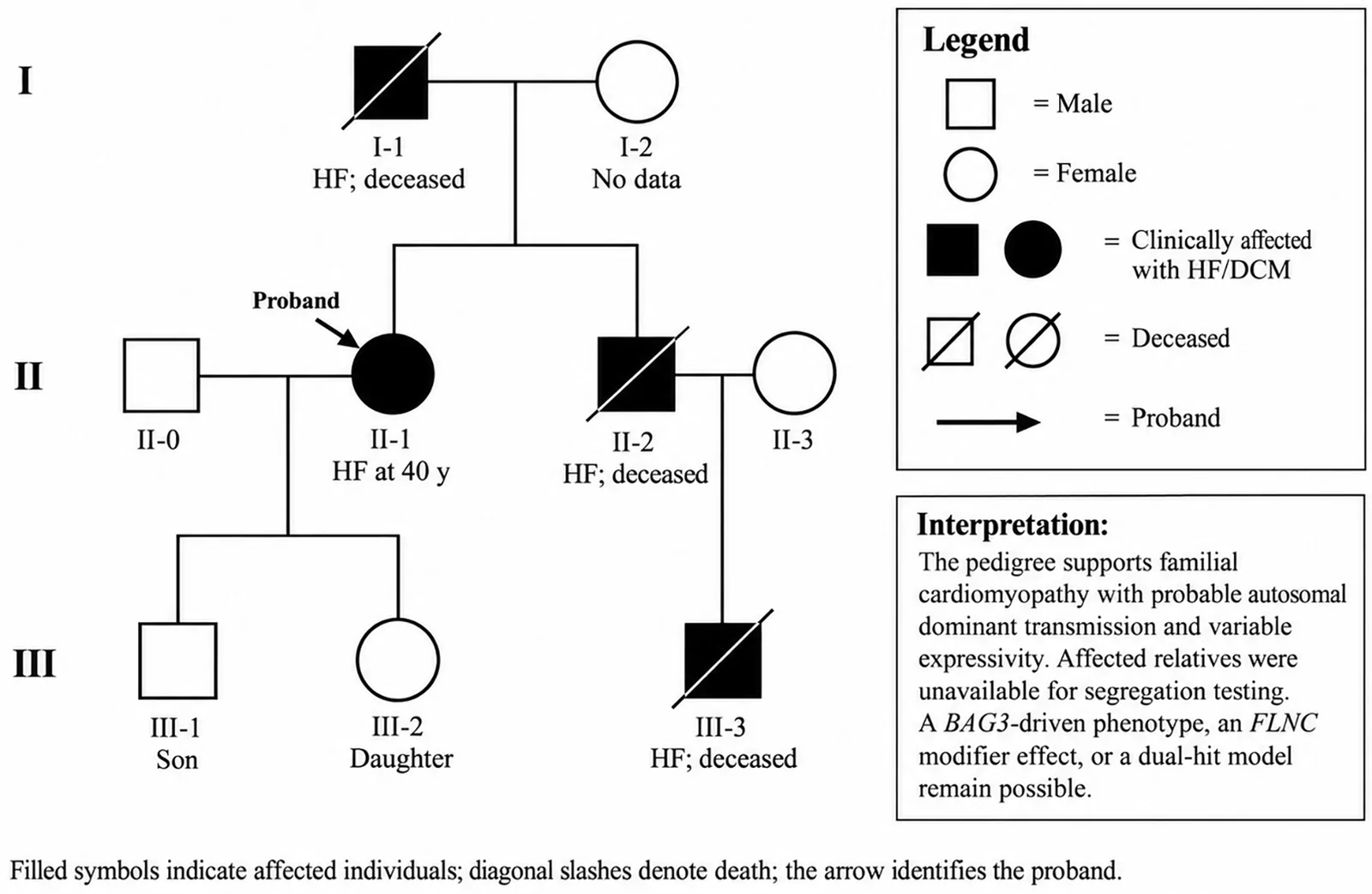
Pedigree of the family with heart-failure-related deaths and available genetic results. Filled symbols indicate clinically affected individuals; diagonal slashes indicate deceased individuals; the arrow identifies the proband. The pedigree is compatible with probable autosomal dominant inheritance with variable expressivity, although genetic testing was unavailable for the deceased affected relatives.

The pedigree is compatible with probable autosomal dominant inheritance, but affected relatives were deceased and unavailable for testing. The BAG3 variant was present in the proband and her daughter, whereas FLNC was absent in both tested children. A modifier role for FLNC in the proband remains unproven.

Filled symbols indicate affected individuals; diagonal slashes denote death; the arrow identifies the proband. To minimize identifiability, only generalized family-history information is shown for deceased relatives; no genetic data, clinical images, or medical-record extracts from those relatives are presented. Genetic findings are summarized in Table 2 and Supplementary Table S6.

**Table 2:** Genetic findings and family validation.

Gene/region	Variant	Zygosity/burden	Classification	Family validation	Clinical interpretation
BAG3	NM_004281.4:c.180 + 1G > T	Heterozygous	Likely pathogenic	Daughter: variant-positive; son: wild-type	Canonical splice-site loss-of-function variant consistent with BAG3-related autosomal dominant DCM. No prior report was identified in ClinVar, dbSNP, gnomAD, or the published literature on reassessment; described here as previously unreported.
FLNC	NM_001458.5:c.761_779del, p.(Leu254Profs*14)	Heterozygous	Likely pathogenic	Daughter: wild-type; son: wild-type	Proband-specific frameshift loss-of-function variant; clinically relevant as a possible risk marker or modifier, but segregation with disease is unproven. Previously reported in a Chinese cardiomyopathy cohort and therefore not novel (11).
MT-ND4	NC_012920.1:m.11778G > A	34.4% heteroplasmy	Pathogenic	Both children variant-positive	Primarily associated with Leber hereditary optic neuropathy; cardiac contribution unconfirmed.
16p13.11 duplication	seq[hg38]16p13.11 (14702996_16393923)x3	Copy-number gain approximately 1.69 Mb	Variant of uncertain significance	No family testing performed	Requires phenotype correlation and segregation analysis; no confirmed cardiomyopathy linkage.

### Genetic findings

Enhanced whole-exome sequencing identified heterozygous likely pathogenic variants in BAG3 (NM_004281.4:c.180 + 1G > T) and FLNC [NM_001458.5:c.761_779del, p.(Leu254Profs*14)]. The laboratory used targeted capture, high-throughput sequencing, and Sanger validation of selected familial variants (Figure 3).

**Figure 3:**
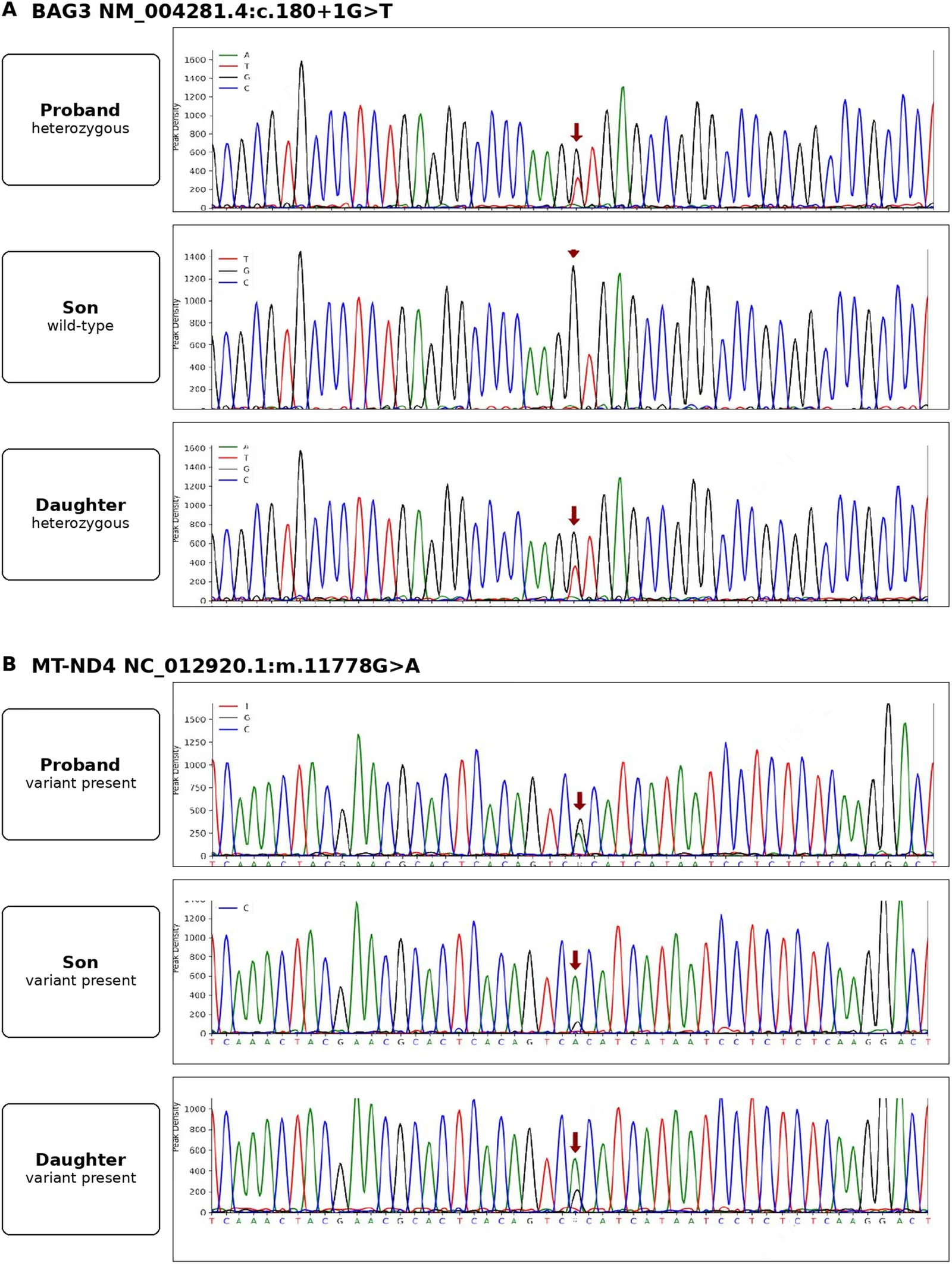
De-identified Sanger sequencing chromatograms for BAG3 and MT-ND4. (A) BAG3 NM_004281.4:c.180+1G>T was detected heterozygously in the proband and her daughter but not in her son. (B) MT-ND4 NC_012920.1:m.11778G>A was detected in the proband and both children. Red arrows indicate the variant positions. Sample identifiers were removed.

The BAG3 canonical splice-site variant was absent from gnomAD and classified using PM2_P and PVS1. BAG3-related DCM is mediated by haploinsufficiency (ClinGen haploinsufficiency score 3). On reassessment of major public resources, including ClinVar, dbSNP, gnomAD, and the published literature, we did not identify a prior report of BAG3 NM_004281.4:c.180 + 1G > T; therefore, we describe this variant as previously unreported rather than definitively novel. The daughter was heterozygous and the son was wild-type.

The FLNC frameshift variant was absent from gnomAD and predicted to cause loss of function, a recognized mechanism in FLNC-associated cardiomyopathy. Importantly, FLNC NM_001458.5:c.761_779del, p.(Leu254Profs*14) has been previously reported in a Chinese cardiomyopathy cohort and was classified as likely pathogenic; therefore, this FLNC variant is not novel (11). Both children were wild-type; therefore, segregation with disease could not be established. Additional findings were heteroplasmic MT-ND4 m.11778G > A (34.4%), classified as pathogenic, and a 16p13.11 duplication classified as a variant of uncertain significance. Both children carried MT-ND4. These findings were not considered established explanations for the cardiac phenotype.

Overall, the data support an inherited cardiomyopathy background but do not establish a single-gene causal mechanism. The BAG3 variant is a plausible familial contributor because it was identified in the proband and daughter and is consistent with an established loss-of-function mechanism in autosomal dominant DCM. The FLNC truncating variant may also contribute to the proband's fibrotic and arrhythmic phenotype. Because affected deceased relatives were unavailable for testing, the relative contribution of BAG3 and FLNC cannot be definitively determined.

## Discussion

The acute heart failure episode occurred after an upper respiratory tract infection, raising an initial clinical question of inflammatory myocardial injury. However, serial cardiac troponin I and myocardial enzyme measurements remained within the reference ranges, and CMR did not include dedicated inflammatory sequences such as T2-weighted imaging, T2 mapping, or STIR. Accordingly, active myocarditis cannot be established from the available data. The temporal association with infection is more appropriately interpreted as a possible trigger for acute decompensation that revealed an underlying genetically susceptible myocardium, rather than as proof of acute myocarditis. Reports of inflammatory or myocarditis-like presentations in carriers of truncating FLNC variants remain relevant as a phenotypic context but should not be extrapolated to a definitive diagnosis in this patient (7).

Coexisting likely pathogenic BAG3 and FLNC variants appear uncommon. BAG3- and FLNC-related cardiomyopathies are well described separately (3, 4, 6, 8), whereas only 2.3% of genotype-positive patients in one DCM cohort carried a second likely pathogenic/pathogenic variant in another robust DCM gene (10). The two variants in the present proband do not have the same novelty status: BAG3 c.180 + 1G > T appears previously unreported based on our reassessment of major public databases and the literature, whereas FLNC c.761_779del, p.(Leu254Profs*14) was reported previously in a Chinese cardiomyopathy cohort (11). A targeted search of PubMed, Web of Science, CNKI, Wanfang, and the Chinese Biomedical Literature Database through July 2026 did not identify a comparable Chinese case with both loss-of-function variants and family Sanger validation (12). This case is therefore hypothesis-generating, not evidence of dual-gene causality.

The BAG3 variant represents a plausible familial contributor because loss-of-function variants are an established cause of autosomal dominant DCM and the splice-site variant was transmitted to the daughter. However, the contribution of the FLNC truncating variant cannot be excluded. Truncating FLNC variants are associated with myocardial fibrosis and ventricular arrhythmias (6, 8), features that overlap with the proband's non-ischemic LGE and ventricular ectopy. Because the deceased affected relatives were unavailable for genetic testing, the available family data do not permit assignment of predominant causality to either gene. Persistent LVEF reduction, LGE, and ventricular ectopy justify continued arrhythmic surveillance.

The pedigree supports familial disease but cannot distinguish among a BAG3-associated phenotype, a clinically relevant FLNC contribution, or an oligogenic/double-hit context. DNA from the deceased father, brother, and nephew was unavailable, preventing formal segregation analysis and limiting genotype-phenotype attribution.

At 2 months, symptomatic and biomarker improvement occurred without meaningful reverse remodeling: LVEF remained 25%. Symptomatic hypotension limited sacubitril/valsartan, so any low-dose re-challenge should follow achievement of euvolemia and stable blood pressure while preserving tolerated beta-blocker, mineralocorticoid receptor antagonist, and sodium-glucose cotransporter 2 inhibitor therapy. QTc prolongation and ventricular premature complexes warrant correction of reversible factors, avoidance of QT-prolonging drugs, electrolyte optimization, and ambulatory rhythm monitoring when feasible.

Given persistent LVEF ≤35%, non-ischemic LGE, ventricular ectopy, a truncating FLNC variant, and an adverse family history, early multidisciplinary evaluation for primary-prevention implantable cardioverter-defibrillator (ICD) therapy is appropriate rather than relying solely on a routine waiting period for LVEF recovery. ICD implantation was discussed with the patient after individualized assessment of potential arrhythmic risk and preventive benefit; however, she did not proceed with implantation because of personal socioeconomic considerations. Continued optimization of tolerated therapy, ambulatory rhythm monitoring when feasible, and repeated device-risk assessment remain necessary (1, 2, 6, 8).

The genotype-positive daughter requires developmentally appropriate genetic counseling and periodic clinical assessment, ECG, and echocardiography, with CMR or ambulatory monitoring if symptoms or abnormalities develop. The son's negative BAG3 and FLNC results reduce, but do not eliminate, concern because segregation in affected relatives was unavailable. Both children should receive counseling regarding MT-ND4, which is primarily associated with Leber hereditary optic neuropathy.

## Limitations

Endomyocardial biopsy was not performed. Serial cardiac troponin I and myocardial enzyme measurements remained within reference ranges, while CMR lacked dedicated inflammatory sequences (T2-weighted imaging, T2 mapping, and STIR). Therefore, active myocarditis could not be confirmed or completely excluded, and the infection-associated presentation should be interpreted cautiously.

DNA samples from deceased affected relatives were unavailable, preventing formal segregation analysis and limiting assessment of the relative contributions of BAG3 and FLNC or a potential oligogenic/double-hit effect.

Medium- and long-term follow-up of the proband and genotype-positive daughter are not yet available.

The MT-ND4 and 16p13.11 findings lack clear cardiac phenotype correlation and cannot be reliably implicated in the DCM phenotype based on current data.

Standard 24-hour ambulatory Holter monitoring was not performed during admission because of local reimbursement restrictions; continuous bedside telemetry was used, but quantitative 24-hour ventricular ectopy burden could not be calculated.

## Learning points

An acute heart failure episode following infection may reveal previously unrecognized inherited cardiomyopathy; infection-associated presentations should not be equated with confirmed myocarditis when biomarkers and inflammatory CMR criteria are not supportive.

Coexisting cardiomyopathy-associated variants require cautious genotype-phenotype interpretation and should not be assumed to establish dual-gene causality without segregation or functional evidence.

Truncating FLNC variants, non-ischemic LGE, ventricular ectopy, persistent severe LV dysfunction, and adverse family history should prompt structured arrhythmic risk assessment and individualized consideration of primary-prevention ICD therapy.

Genotype-positive children require age-appropriate longitudinal cardiac surveillance and genetic counseling (13).

## Conclusions

This case supports complex familial non-ischemic DCM with coexisting likely pathogenic BAG3 and FLNC variants whose relative phenotypic contributions remain uncertain. The infection-associated acute decompensation was not accompanied by elevation of cardiac troponin I or myocardial enzymes, and active myocarditis could not be confirmed because dedicated inflammatory CMR sequences were unavailable. Persistent severe dysfunction, non-ischemic LGE, ventricular ectopy, and the FLNC truncating variant emphasize the need for optimized tolerated therapy, structured arrhythmic and device-risk assessment, and cascade surveillance of genotype-positive relatives.

## Data Availability

The datasets presented in this study can be found in online repositories. The names of the repository/repositories and accession number(s) can be found in the article/Supplementary Material.

## References

[1] Arbelo E Protonotarios A Gimeno JR Arbustini E Barriales-Villa R Basso C. 2023 ESC guidelines for the management of cardiomyopathies. Eur Heart J. (2023) 44(37):3503–626. 10.1093/eurheartj/ehad194 37622657

[2] Heidenreich PA Bozkurt B Aguilar D Allen LA Byun JJ Colvin MM. 2022 AHA/ACC/HFSA guideline for the management of heart failure. Circulation. (2022) 145(18):e895–e1032. 10.1161/CIR.0000000000001063 35363499

[3] Qu HQ Feldman AM Hakonarson H. Genetics of BAG3: a paradigm for developing precision therapies for dilated cardiomyopathies. J Am Heart Assoc. (2022) 11(23):e027373. 10.1161/JAHA.122.027373 36382946 PMC9851466

[4] Newman NA Burke MA. Dilated cardiomyopathy: a genetic journey from past to future. Int J Mol Sci. (2024) 25(21):11460. 10.3390/ijms252111460 39519012 PMC11546582

[5] Oliveira GMM Fontes-Carvalho R Cardim N Rochitte CE. Challenges and applications of genetic testing in dilated cardiomyopathy: genotype, phenotype and clinical implications. Arq Bras Cardiol. (2023) 120(10):e20230342. 10.36660/abc.20230342 38055534 PMC10697682

[6] Gigli M Stolfo D Graw SL Merlo M Gregorio C Nee Chen S. Phenotypic expression, natural history, and risk stratification of cardiomyopathy caused by filamin C truncating variants. Circulation. (2021) 144(20):1600–11. 10.1161/CIRCULATIONAHA.121.053521 34587765 PMC8595845

[7] Vrettos A Demetriades P Ortiz M Domínguez F García-Pavía P Suárez-Mier MP. Pathogenic truncating filamin C mutations presenting as acute myocarditis: a case series with insights from cardiac magnetic resonance and histological analysis. Eur Heart J Case Rep. (2024) 8(3):ytae111. 10.1093/ehjcr/ytae111 38476289 PMC10928485

[8] Filamin C Registry Consortium, Gigli M Stolfo D Barbati G Graw S Chen SN. Arrhythmic risk stratification of carriers of filamin C truncating variants. JAMA Cardiol. (2025) 10(4):359–69. 10.1001/jamacardio.2024.5543 39937464 PMC11822610

[9] Sorella A Galanti K Iezzi L Gallina S Mohammed SF Sekhri N. Diagnosis and management of dilated cardiomyopathy: a systematic review of clinical practice guidelines and recommendations. Eur Heart J Qual Care Clin Outcomes. (2025) 11(2):206–16. 10.1093/ehjqcco/qcae109 39674807 PMC11879293

[10] Stroeks SLVM Lunde IG Hellebrekers DMEI Claes GRF Wakimoto H Gorham J. Prevalence and clinical consequences of multiple pathogenic variants in dilated cardiomyopathy. Circ Genom Precis Med. (2023) 16(2):e003788. 10.1161/CIRCGEN.122.003788 36971006

[11] Xing G Chen L Lv L Hu C Liu S Duan Y. Genetic and clinical characterization of FLNC variants in Chinese patients with cardiomyopathy. J Cardiovasc Dev Dis. (2025) 12(12):492. 10.3390/jcdd12120492 41440871 PMC12734206

[12] Li XJ Zhou NW Sun MM Li W Wang YS Xie HL. Pedigree study of FLNC variants in Chinese han patients with cardiomyopathy. Fudan Univ J Med Sci. (2021) 48(5):586–91. 10.3969/j.issn.1672-8467.2021.05.003

[13] Parker LE Landstrom AP. The clinical utility of pediatric cardiomyopathy genetic testing. Children (Basel). (2021) 8(11):1039. 10.3390/children8111039 34776723 PMC8579834

