# Supplementary material for: Familial severe non-ischemic dilated cardiomyopathy with coexisting likely pathogenic BAG3 and FLNC variants: a case report
Source: Front Cardiovasc Med. 2026 Oct 30;13:2000926. doi: 10.3389/fcvm.2026.2000926 (PMC13759278; doi:10.3389/fcvm.2026.2000926)
Supplement: Supplementary file 1 [file Datasheet1.pdf]

Supplementary Materials

Supplementary clinical clarification: Serial cardiac troponin I and myocardial enzyme measurements obtained during hospitalization remained within their respective reference ranges. Dedicated inflammatory CMR sequences, including T2-weighted imaging, T2 mapping, and STIR, were not acquired; therefore, active myocarditis could not be confirmed from the available CMR examination.

Supplementary Table 1. Serial 12-lead electrocardiographic parameters

Standard 24-hour ambulatory Holter monitoring was not performed due to local medical insurance policies; simultaneous telemetry and Holter would lead to full non-reimbursement of cardiac monitoring fees, only bedside telemetry was available. Ventricular premature complexes were documented on available ECG and bedside telemetry; however, a quantitative 24-hour ventricular ectopic burden was not available.

Test timepoint	Baseline rhythm	Resting HR (bpm)	PR interval (ms)	QRS duration (ms)	QTc (ms)	ST/T-wave abnormalities	Arrhythmia finding
Hospital day 1 (admission)	Sinus rhythm	92	169	99	494	QS complexes in V1-V3; diffuse T-wave inversion in V5-V6	Frequent ventricular premature contractions captured by ward telemetry
Hospital day 3 (April 2, 2026)	Sinus rhythm	69	174	95	463	QS complexes in V1-V3; T-wave inversion in V4-V6	No ventricular ectopy detected on available 12-lead ECG; bedside telemetry continued
2-month outpatient follow-up	Sinus rhythm	68	165	115	479	QS complexes in V1-V3; T-wave inversion in V5-V6	Occasional ventricular premature contractions on ECG

Supplementary Table 2. Serial quantitative transthoracic echocardiographic parameters

Normal reference range for adult female: LVEDD <50 mm; LVEF >=55%; interventricular septum / left ventricular posterior wall thickness 6-11 mm; LAD <40 mm; RVEDD <30 mm; estimated PASP <35 mmHg. N/A = data not recorded in original medical records.

Imaging timepoint	LVEDD (mm)	LVESD (mm)	LVEF (%)	IVS thickness (mm)	LVPW thickness (mm)	LAD (mm)	RVEDD (mm)	Estimated PASP (mmHg)	Valvular regurgitation	Pericardial effusion
Within 24h of admission	69	N/A	21	9	8	42	26	22	Mild-to-moderate MR; mild TR; mild AR	Small
Pre-discharge (April 10)	N/A	N/A	27	8	7	42	26	28	Mild MR	Small
2-month outpatient follow-up	N/A	N/A	25	7	7	39	24	24	Mild MR	Small

Supplementary Table 3. Guideline-directed medical therapy: titration, hemodynamic response, and adverse events

ARNI = angiotensin receptor-neprilysin inhibitor; MRA = mineralocorticoid receptor antagonist; SGLT2i = sodium-glucose cotransporter 2 inhibitor; PO = oral; IV = intravenous; QD = once daily; BID = twice daily; SBP = systolic blood pressure; HR = heart rate; N/A = no standardized target dose.

Drug class	Generic name	Initial dose and route	Titration course	Guideline target dose	Discharge dose	2-month follow-up dose	Stable HR (bpm)	Stable SBP (mmHg)	Adverse events	Overall tolerability
ARNI	Sacubitril/valsartan	100 mg PO QD	Discontinued on hospital day 1 because of symptomatic hypotension; later re-challenged/restarted at low outpatient dose when hemodynamics allowed	200 mg PO BID	Discontinued	50 mg PO QD	68	100	Symptomatic hypotension during index admission	Blood-pressure limited
Beta-blocker	Metoprolol succinate	47.5 mg PO QD	No dose uptitration implemented	190 mg PO QD	47.5 mg PO QD	47.5 mg PO QD	68	100	None	Good
MRA	Spironolactone	20 mg PO QD	No dose uptitration implemented	25-50 mg PO QD	20 mg PO QD	20 mg PO QD	68	100	None	Good
SGLT2 inhibitor	Dapagliflozin	10 mg PO QD	No dose uptitration implemented	10 mg PO QD	10 mg PO QD	10 mg PO QD	68	100	None	Good
Loop diuretic	Furosemide	10 mg IV QD	Switched to oral formulation without further adverse events	N/A	20 mg PO QD	20 mg PO QD	68	100	None	Good

Supplementary Table 4. Serial serum renal function and electrolyte profiles

Sampling timepoint	Creatinine (umol/L)	eGFR (mL/min/1.73 m^2)	BUN (mmol/L)	Serum K^+ (mmol/L)	Serum Na^+ (mmol/L)	Serum Cl^- (mmol/L)	Clinical laboratory context
Admission baseline	69	95.54	5.83	3.36	143	105	Baseline organ function assessment prior to GDMT initiation
Hospital day 6	56	112.43	4.80	3.81	138	101	Serial monitoring during diuretic and neurohormonal antagonist titration
Pre-discharge	N/A	N/A	N/A	3.73	138	105	Safety evaluation before hospital discharge
2-month outpatient follow-up	46	119.94	5.68	4.22	139	105.9	Long-term safety surveillance of chronic heart failure medications

Supplementary Table 5. Genetic findings and family validation results

Gene/region	Variant nomenclature	Zygosity / burden	ACMG classification	Family Sanger validation	Clinical phenotype relevance
BAG3	NM_004281.4:c.180+1G>T	Heterozygous	Likely pathogenic	Daughter: variant-positive; son: wild-type	Canonical splice-site loss-of-function variant consistent with BAG3-related autosomal dominant DCM. Its familial relevance is supported by detection in the proband and daughter, although segregation in deceased affected relatives was unavailable.
FLNC	NM_001458.5:c.761_779del, p.(Leu254Profs*14)	Heterozygous	Likely pathogenic	Daughter: wild-type; son: wild-type	Truncating loss-of-function variant associated with dilated/arrhythmogenic cardiomyopathy, myocardial fibrosis, and increased ventricular arrhythmic risk. Disease segregation could not be established because affected deceased relatives were unavailable for genetic testing.
MT-ND4	NC_012920.1:m.11778G>A	34.4% heteroplasmy	Pathogenic	Both children variant-positive	Primarily associated with Leber hereditary optic neuropathy; cardiac phenotypic contribution unconfirmed.
16p13.11 duplication	seq[hg38]16p13.11(14702996_16393923)x3	~1.69 Mb copy-number gain	Variant of uncertain significance	No family testing performed	Requires phenotype correlation and segregation analysis; no confirmed cardiomyopathy linkage.

Supplementary Table 6. Clinical phenotype and genotype summary for all pedigree members Subject identifiers correspond to the revised pedigree in Figure 2.

Subject ID	Family relationship	Clinical cardiac phenotype	Genetic variant status
I-1	Proband's father	Reported adult-onset heart failure; deceased	Genetic testing unavailable
I-2	Proband's mother	Clinical cardiac phenotype unavailable	Genetic testing not performed
II-0	Proband's spouse	Clinical cardiac phenotype unavailable	Genetic testing not performed
II-1	Proband (index case)	Severe non-ischemic DCM with decompensated heart failure at age 40	BAG3+, FLNC+, MT-ND4+
II-2	Proband's younger brother	Reported adult-onset heart failure; deceased	Genetic testing unavailable
II-3	Younger brother's spouse	Clinical cardiac phenotype unavailable	Genetic testing not performed
III-1	Proband's son	No reported heart failure or DCM to date	BAG3-, FLNC-, MT-ND4+
III-2	Proband's daughter	No reported heart failure or DCM to date	BAG3+, FLNC-, MT-ND4+
III-3	Nephew (younger brother's son)	Reported childhood-onset heart failure; deceased	Genetic testing unavailable
